# A case of disseminated nocardia infection with initial symptoms manifesting as cognitive impairment: Case report and literature review

**DOI:** 10.1097/MD.0000000000039535

**Published:** 2024-12-06

**Authors:** Xiayahu Li, Weiguo Zhou, Kai Zhao, Yaolin Li

**Affiliations:** a Department of Critical Care Medicine, Chengdu Second People’s Hospital, Chengdu, China; b Department of Critical Care Medicine, The Fourth People’s Hospital of Chengdu, Chengdu, China; c Department of Pulmonary and Critical Care Medicine, The Third People’s Hospital of Chengdu, Chengdu, China.

**Keywords:** antimicrobial therapy, diagnosis, mortality, next-generation sequencing (NGS), Nocardia, opportunistic infection

## Abstract

**Rationale:**

*Nocardia* infections, although rare, pose significant challenges in diagnosis and treatment, especially when involving the central nervous system (CNS). Mortality rates in such cases can be high, highlighting the need for early recognition and tailored antimicrobial therapy.

**Patient concerns:**

A 58-year-old male with a history of chronic obstructive pulmonary disease, antineutrophil cytoplasmic antibody–associated glomerulonephritis, and steroid-induced diabetes mellitus presented with disorganized speech, fever, cough, dyspnea, and psychiatric symptoms.

**Diagnoses:**

The patient was diagnosed with severe pneumonia, left pneumothorax, bilateral pulmonary bullae, and CNS involvement. Next-generation sequencing (NGS) identified *Nocardia farcinica* as the causative agent.

**Interventions:**

Initial treatment with ceftriaxone was ineffective. Upon identification of *N. farcinica* via NGS, the patient was started on a tailored antimicrobial regimen consisting of sulfamethoxazole, linezolid, and meropenem.

**Outcomes:**

Despite initial clinical improvement, the patient was discharged early due to financial constraints. Unfortunately, he later succumbed to the infection.

**Lessons:**

This case underscores the difficulty of diagnosing *Nocardia* infections, particularly when they involve the CNS. The use of advanced diagnostic tools such as NGS, along with early and appropriate antimicrobial therapy, is crucial for improving patient outcomes. Financial and healthcare access challenges may impact the success of treatment, emphasizing the importance of comprehensive follow-up and patient support.

## 1. Introduction

Edmond Nocardia, a veterinarian, was the first to isolate Nocardia from bovine lymphadenitis in 1888.^[1]^ However, to date, Nocardia has received relatively little attention as a human pathogen. It is an aerobic, Gram-positive, filamentous bacterium that is widely present in water and soil. It is a rare opportunistic infection, and nocardiosis usually occurs in patients with cell-mediated immune suppression, although occasionally it can also affect immunocompetent patients.^[2]^ Routine examination methods include direct smear microscopy and culture. Nocardia multiply slowly, with colonies usually visible in 2 to 7 days and sometimes it may take up to 4 weeks, especially when growing on selective mycobacterial media (Koneman’s color atlas and textbook of diagnostic microbiology). Second-generation sequencing of the macrogenome (mNGS) has the advantages of full coverage, short time, and no bias in the detection of pathogenic microorganisms in specimens. The mNGS technique has been widely used for the clinical pathogenic diagnosis of infectious diseases and a number of studies have been reported.^[3–5]^ The incidence of nocardial disease has been increasing year by year, which is considered to be related to the improvement of laboratory identification and the increase of high-risk groups.^[6]^ Nevertheless, the management of this infectious disease is a challenge in clinical practice, and the mortality rate for patients with disseminated infection can reach 20% to 30%, rising to 50% if the central nervous system is involved.^[7]^

Nocardia infections are characterized by their rarity, episodic nature, and insidious onset, often presenting with nonspecific clinical symptoms.^[8]^ The most common clinical manifestations include respiratory and systemic symptoms such as fever, cough, headache, sputum production, dyspnea, malaise, weight loss, and shortness of breath. Previously, the lungs and brain have been identified as the primary sites of infection, accounting for 62% to 86% of cases in the lungs^[9–12]^ and 11% to 44% in the brain.^[9,13,14]^ Among brain involvement, impaired consciousness is the predominant manifestation, while mental changes as the initial symptom are infrequent.

Diagnosing Nocardia infections has traditionally been challenging due to nonspecific clinical presentations and the slow growth of the bacterium in culture. Traditional diagnostic methods, such as smear microscopy and culture, rely on visualizing and growing the pathogen, but these processes can be time-consuming and may delay appropriate treatment. Smear microscopy has limited sensitivity, especially when the pathogen is present in low numbers in the specimen, leading to potential false negatives. While culture is considered the gold standard, Nocardia’s slow growth often means that results can take weeks to obtain. This delay can negatively impact patient outcomes, as treatment may initially be empirical and possibly ineffective without a definitive diagnosis.

In contrast, next-generation sequencing (NGS) has revolutionized the diagnostic landscape. NGS offers a rapid, comprehensive, and unbiased method for detecting a wide range of pathogens, including Nocardia species. It can identify trace amounts of pathogen DNA in clinical samples, even for organisms that are difficult to culture using traditional methods. This technology not only reduces the time to diagnosis but also enhances the accuracy and breadth of pathogen detection, allowing clinicians to initiate targeted antimicrobial therapy sooner. The role of NGS is particularly crucial in cases with atypical presentations or resistance to conventional antibiotics, highlighting its importance in the clinical management of these complex infections.

## 2. Pre-hospital performance and treatment

A 58-year-old male was admitted to the hospital on December 26, 2021, due to disorganized speech. Six days before admission, the patient experienced pyrexia (maximum temperature of 38.5°C) accompanied by cough and dyspnea. The patient self-administered “Lianhuaqingwen, Fufang Fenkawei Majia Capsules, Furtding Oral Solution, and Ibuprofen Oral Solution” resulting in antipyresis but aggravated cough and dyspnea. Two days before admission, the patient presented with psychiatric and cognitive manifestations including emotional irritability, persecutory delusions, hallucinations, and insomnia. A CT scan conducted in a local hospital revealed left pneumothorax with 40% compression, bilateral pulmonary bullae, and chronic infection manifestations. A mass was identified in the lower lobe of the left lung. Laboratory assessments demonstrated leukocytosis (white blood cell count of 6.49 × 10^9^/L, neutrophil ratio of 94.5%), anemia (hemoglobin level of 85 g/L), and elevated levels of C-reactive protein (245.21 mg/L). Ceftriaxone was administered to the patient, but the therapeutic response was suboptimal. The patient subsequently exhibited emotional lability, visual hallucinations, and auditory hallucinations.

## 3. Past medical history

The patient’s medical history includes chronic obstructive pulmonary disease, ANCA-associated glomerulonephritis, renal anemia, steroid-induced diabetes mellitus, and hypertension. In the 3 months before admission, the patient received pulse therapy for ANCA-associated glomerulonephritis (methylprednisolone 240 mg for 3 days followed by methylprednisolone 80 mg for 3 days) and was treated with cyclophosphamide (800 mg for 3 days) most recently on December 15, 2021.

The patient was taking prednisolone acetate 40 mg once daily, famotidine 20 mg once daily, alfacalcidol 0.25 μg once daily, amlodipine besylate and benazepril hydrochloride 5 mg twice daily, irbesartan 75 mg once daily, liraglutide 1 mg twice daily, calcium carbonate and vitamin D3 1 tablet once daily, and atorvastatin calcium 10 mg once at night before admission. Other past medical history includes pneumothorax 5 years ago, left eye blindness 2 years ago, and appendicitis 4 months ago. The patient has a 20-year history of smoking, approximately 40 to 60 cigarettes per day.

The patient worked as a quarryman in a dusty environment and imaging studies suggested silicosis, although it was not confirmed. The patient has no significant family history.

## 4. Condition at admission

On admission, the patient had elevated blood pressure, tachycardia, and dyspnea, but no fever was observed (Table 1). Physical examination revealed the patient’s agitation, delusions, and hallucinations. The pupils were symmetrical, approximately 3mm in size, and responsive to light reflex. Barrel chest changes were observed and the trachea was deviated to the right. The left upper lung had no breath sounds, while both lower lungs had diminished breath sounds and fine wet rales bilaterally. No edema was present. Other examinations were challenging due to the patient’s agitation. Laboratory tests showed elevated levels of certain markers of infection, renal function, and cardiac function (Table 1).

**Table 1:** Vital signs and laboratory tests of patients on admission

Clinical		Laboratory variable		
Variable	Measurements	Variable	Measurements	Normal value
Temperature	36.9°C	WBC	8.85 × 10^9^/L	3.5–9.5 × 10^9^/L
Blood pressure	152/102 mm Hg	NE#	8.21 × 10^9^/L	1.8–6.3 × 10^9^/L
Respiratory rate	30 breaths/min	LY#	0.26 × 10^9^/L	1.1–3.2 × 10^9^/L
Heart rate	156 beats/min	NEut%	92.8	40–75
Oxygen saturation	99%	LYMPH%	3.0	20–50
		Haemoglobin	125 g/L	120–160 g/L
		Hematocrit%	38	37–47
		Platelets	298 × 10^9^/L	100–300 × 10^9^/L
		C-reactive protein	244.42 mg/L	<10 mg/L
		PCT	6.462 ng/mL	0–0.05 ng/mL
		BUN	24.5 mmol/L	1.43–714 mmol/L
		Cr-S	257 mmol/L	44–92 mmol/L
		BNP	197pg/mL	<100 pg/mL
		G test [(1-3)-β-D-Glucan Assay]	37.5 pg/mL	<70 pg/mL

BNP = brain natriuretic peptide, BUN = blood urea nitrogen, Cr-S = serum creatinine, LY# = lymphocyte count, LYMPH% = percentage of lymphocytes, NE# = neutrophil count, NEut% = percentage of neutrophils, PCT = procalcitonin, WBC = white blood cell.

## 5. Treatment and examination results during hospitalization

**Figure 1.**
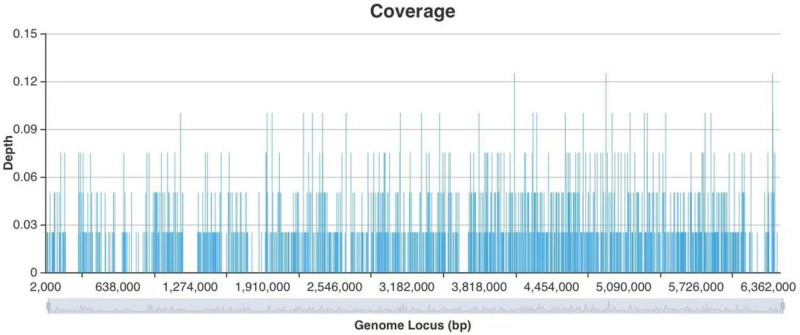
Sample NGS gene sequencing results. NGS = next-generation sequencing.

**Figure 2.**
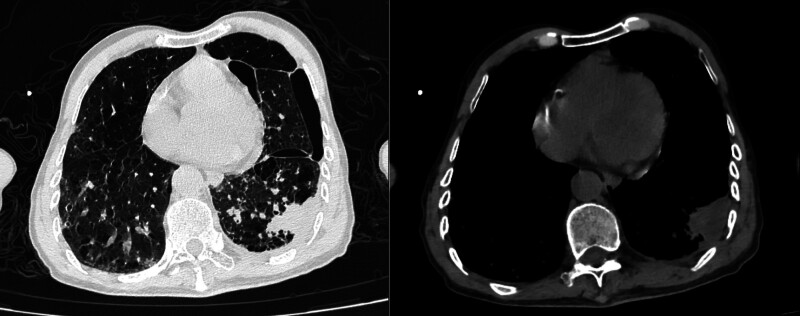
Chest CT, the anteroposterior diameter of the chest is increased. In the left lung, there is a patchy area without lung markings with fluid density and a surrounding edge of compressed lung tissue, resulting in approximately 40% lung collapse. Both lungs show increased and disordered lung markings, decreased lung field density, and multiple cystic areas without lung markings, as well as scattered slightly elevated strip-like densities. An irregular mass-like density with uneven internal density and a maximum transverse section of about 44 mm * 30 mm is present in the basal segment of the lower lobe of the left lung, with adjacent pleural thickening and adhesion. Small patchy shadows are seen in the proximal lumen of the trachea and right upper lobe bronchus. Both pleurae are thickened, and there is a small amount of fluid in the left pleural cavity. Enlarged lymph nodes are seen in the mediastinum.

**Figure 3.**
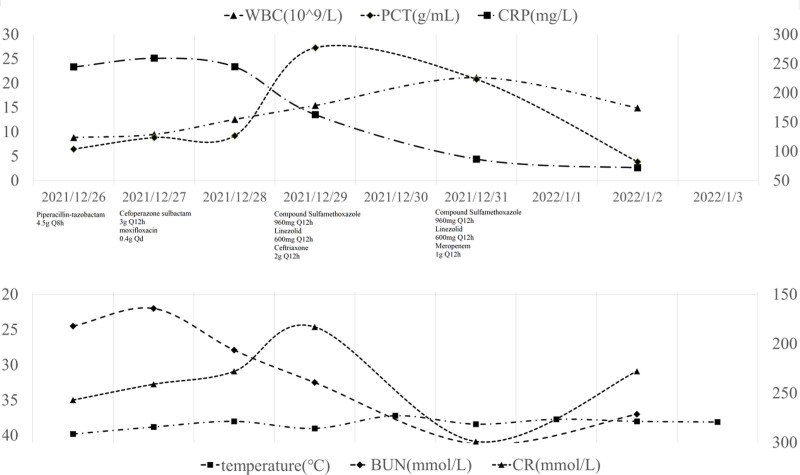
Laboratory indicators and anti-bacterial drug regimen. BUN = blood urea nitrogen, CR = creatinine, CRP = C-reactive protein, PCT = procalcitonin, WBC = white blood cell count.

Upon admission, the patient was diagnosed with severe pneumonia and was started on empirical antibacterial therapy with piperacillin-tazobactam at a dosage of 4.5 g every 8 hours, along with thoracentesis for decompression. However, the patient subsequently developed a high fever, with a maximum temperature of 39.8°C, and drowsiness, leading to transfer to the intensive care unit on December 27, 2021. Upon admission to the intensive care unit, blood and sputum samples were collected for culture, and peripheral blood, cerebrospinal fluid, and pleural fluid were collected for next-generation sequencing (NGS) analysis. The patient’s clinical features and laboratory indicators were closely monitored. The cerebrospinal fluid was clear, colorless, and had notable white precipitation after standing. The cerebrospinal fluid pressure was measured at 280 mm H_2_O. The cerebrospinal fluid laboratory test results are shown in Table 2. The pleural effusion was brown and odorless. Due to the limited sample size, NGS analysis was only performed on the pleural effusion. The NGS examination results and CT scan findings are presented in Figures 1 and 2, respectively. After admission to the intensive care unit, the patient’s antibacterial therapy was adjusted to cefoperazone-sulbactam combined with moxifloxacin. On December 29, 2021, NGS results suggested an infection with Nocardia farcinica, prompting a clinical pharmacist to assist in adjusting the anti-infective regimen to include trimethoprim-sulfamethoxazole, linezolid, and ceftriaxone. Blood and sputum cultures remained inconclusive up to the patient’s discharge from the hospital. On December 31, 2021, a reassessment of the anti-infective efficacy revealed that the patient’s procalcitonin and C-reactive protein levels had decreased, but the white blood cell count remained elevated. Consequently, the patient was switched to meropenem instead of ceftriaxone for continued anti-infective therapy (Fig. 3).

**Table 2:** Cerebrospinal fluid laboratory test results

Variable	Measurements	Normal value
Adenosine dehydrogenase	18 U/L	≤5 U/L
Glutathione aminotransferase	35 U/L	<20 U/L
Lactate dehydrogenase	2762 U/L	<40/L
Glucose	0.5 mmol/L	2.5–4.4 mmol/L
Total protein	188 mg/dL	15–45 mg/dL
RBC	0 × 10^6^/L	0 × 10^6^/L
WBC	8050 × 10^6^/L	0-8 × 10^6^/L
Leukocyte count	1207.5 × 10^6^/L	NA
Multiple nucleated cells	66842.5 × 10^6^/L	NA

RBC = red blood cell count, WBC = white blood cell count.

## 6. Prognosis and follow-up

Upon monitoring, the patient’s infection indicators showed gradual improvement after December 31, 2021, and the peak temperature exhibited a decreasing trend. However, we regret to report that due to financial reasons, the patient’s family decided to discharge the patient from the hospital without medical intervention or treatment. Unfortunately, we received information through telephone follow-up that the patient passed away approximately 37 hours after leaving the hospital.

## 7. Discussion

Nocardia, primarily an opportunistic pathogen, is most frequently acquired via inhalation, followed by exposure of wounds to the bacteria,^[2]^ and healthcare-associated infections.^[15]^ Patients with compromised immune systems, such as those with autoimmune diseases, use of immunosuppressive agents or glucocorticoids,^[9]^ human immunodeficiency virus infection,^[16]^ trauma,^[17–20]^ as well as those who come into contact with soil or dust (such as horticultural workers)^[21–26]^ or wear contact lenses^[27]^ are commonly affected, facilitating contact with Nocardia. The predominant risk factors for infection in our study were also glucocorticoid and immunosuppressive agent use-induced immunodeficiency, consistent with prior research.^[9]^ In cases without immunocompromise, cardiovascular and respiratory diseases were the most commonly occurring comorbidities.

### 7.1. Nocardia and cognitive impairment

Illustrated by this case, the patient’s history of high-dose glucocorticoid use and occupation as a quarryman suggest immune suppression due to steroid treatment and exposure to Nocardia-containing dust, culminating in respiratory infection and subsequent lung tissue alterations. Nocardia sequences were identified via NGS in sputum and pleural fluid, ultimately disseminating to the brain via blood. The involvement of the central nervous system (CNS) in Nocardia infections can lead to a range of psychiatric and cognitive symptoms, as seen in this case, where psychiatric manifestations such as confusion, disorientation, or behavioral changes may occur due to brain abscesses or other CNS involvement.^[28,29]^ Nocardia infections are rare, sporadic, and insidious. Clinical symptoms lack specificity, and traditional bacterial culture methods may require up to three weeks, further complicated by concurrent bacterial infections. This delay may result in delayed diagnosis and initiation of targeted antimicrobial therapy, potentially fostering disseminated Nocardia infection if initial treatment is inadequate. Given the heightened detection of Nocardia following the widespread adoption of genetic testing, we elected to employ NGS testing, expediting pathogenetic results and facilitating targeted therapy. Further, early identification of CNS involvement is crucial, as it significantly influences the prognosis and therapeutic strategies.^[28,29]^

### 7.2. Gender and age distribution

Most prior studies have reported a higher incidence of Nocardia infection in males than in females, with males comprising approximately 70% of cases.^[14,30,31]^ This observation is attributed to disparities in lifestyle and work habits, leading to heightened exposure risks for males. Nonetheless, a subset of studies has documented an equal distribution of Nocardia infection between genders.^[9,32,33]^ Variations in gender distribution across studies may stem from inclusion criteria based on Nocardia infection with specific comorbidities, influencing gender-specific incidence rates. Additionally, prior studies often lacked robust sample sizes, contributing to divergent findings. Recent investigations have indicated elevated incidence rates of Nocardia infection in the 31 to 40 age group,^[30]^ 61 to 70 age group,^[31]^ and 71 to 80 age group,^[31]^ without a discernible age concentration. Nonetheless, most studies have observed a heightened incidence rate after age 50, consistent with earlier research,^[34]^ suggesting an association between advancing age and heightened Nocardia infection risk. These findings imply that younger age and advanced medical resources may mitigate immune dysfunction occurrences, thereby reducing Nocardia infection incidence.

### 7.3. Treatment options

Primary treatment for Nocardia infection entails antibacterial therapy, with sulfonamide antibiotics, including sulfadiazine and sulfisoxazole, serving as the preferred choice.^[30]^ Additionally, other drugs such as amikacin, imipenem, meropenem, ceftazidime, ceftriaxone, minocycline, moxifloxacin, levofloxacin, linezolid, trimethoprim-sulfamethoxazole (TMP-SMX), ticarcillin-clavulanic acid, and amoxicillin-clavulanic acid, find common use in clinical practice.^[2]^ However, prolonged sulfonamide use may engender TMP-SMX resistance, with peak resistance rates reaching 42%.^[31,32]^ Amikacin, linezolid, and imipenem are also recommended as potential sensitive drugs, boasting sensitivity rates exceeding 50%, corroborating prior studies.^[33,34]^ Consequently, in instances where high-risk populations for Nocardia infection cannot be definitively diagnosed or lack drug sensitivity results, TMP-SMX may be recommended for empirical anti-infection treatment to forestall treatment delays.^[2]^

The development of resistance to TMP-SMX (trimethoprim-sulfamethoxazole) in Nocardia species can be attributed to several mechanisms^[35,36]^: Gene Mutation and Horizontal Gene Transfer: Resistance often arises through mutations in target enzymes dihydropteroate synthase (DHPS) and dihydrofolate reductase (DHFR), which are inhibited by sulfamethoxazole and trimethoprim, respectively. These mutations reduce the binding affinity of the drugs to their targets, rendering them ineffective. Horizontal Gene Transfer: Nocardia species can acquire resistance genes from other bacteria via horizontal gene transfer, which can occur through mechanisms such as conjugation, transformation, or transduction. Selective Pressure: Prolonged and widespread use of TMP-SMX exerts selective pressure on bacterial populations, promoting the survival and proliferation of resistant strains. Factors Limiting Long-Term Use of TMP-SMX,^[37,38]^: Adverse Reactions: TMP-SMX can cause various adverse effects, including hypersensitivity reactions (e.g., rash, Stevens-Johnson syndrome), hematologic abnormalities (e.g., leukopenia, thrombocytopenia), and renal toxicity (e.g., interstitial nephritis). Drug Interactions: TMP-SMX interacts with several other medications, which can exacerbate toxicity or reduce therapeutic efficacy. For instance, concomitant use with warfarin can increase the risk of bleeding, while use with methotrexate can enhance bone marrow suppression. Patient Compliance: The risk of adverse effects and the complexity of managing drug interactions can affect patient compliance, particularly during long-term treatment. Methods for Detecting TMP-SMX Resistance^[39,40]^: Phenotypic Methods: Traditional culture-based methods, such as disk diffusion and broth microdilution, are used to assess the susceptibility of Nocardia isolates to TMP-SMX. These methods, however, are time-consuming and may not always detect resistance mechanisms effectively. Molecular Methods: Polymerase chain reaction (PCR) and sequencing techniques can identify specific resistance genes and mutations associated with TMP-SMX resistance. These methods provide rapid and precise detection compared to phenotypic assays. Next-Generation Sequencing (NGS): NGS offers a comprehensive approach by 3. sequencing the entire genome of Nocardia isolates, identifying all resistance genes and mutations present. This method is becoming more accessible and can provide detailed insights into resistance mechanisms. Therefore, we recommend that patients with conditions improve the monitoring of TMP-SMX resistance during the initial use of TMP-SMX, and patients taking TMP-SMX for a long period of time should be regularly monitored for drug resistance, so as to timely adjust the use of medication and the treatment program to avoid recurrence of the disease. At the same time, during the process of using TMP-SMX, patients should be monitored for adverse drug reactions, and the drug regimen should be adjusted accordingly.

### 7.4. Prognosis

The overall mortality rate of Nocardia infection stands at 11.01%, lower than previously reported rates of 16.2% to 38.2%.^[41–43]^ Elevated mortality rates are often linked to inappropriate antimicrobial treatment stemming from delayed initial diagnosis. Early identification of infection sources and targeted antimicrobial treatment can mitigate mortality associated with disseminated Nocardia infection, with timely employment of sensitive antimicrobial drugs pivotal in reducing mortality. Moreover, managing Nocardia infection is a protracted endeavor, necessitating consistent medication to avert recurrence. Patients utilizing steroids or immunosuppressants necessitate prolonged medication to enhance prognosis.

### 7.5. Impact of financial constraints on patient outcomes

In the discussion of our case study, it’s crucial to consider the broader implications of financial constraints on patient outcomes. Financial barriers significantly impact healthcare access and decisions, especially among low-income populations. For instance, individuals facing financial strain may prioritize basic needs such as food and rent over healthcare expenses, leading to delayed or forgone medical care. This often results in poorer health outcomes and increased emergency room visits, as preventive care and regular checkups are neglected.^[44]^

Research highlights that financial constraints can also cause nonadherence to prescribed treatments. Patients might skip doses or delay purchasing medications due to cost, which can lead to worsened health conditions. Moreover, the stigma associated with discussing financial difficulties can prevent patients from openly communicating these issues with healthcare providers. This can be mistaken for noncompliance, further complicating the patient’s care plan.^[44]^

To mitigate these issues, healthcare providers should take proactive steps to identify and address financial strain in patients. This can include developing cost-effective treatment plans, offering information on financial assistance programs, and fostering a supportive environment where patients feel comfortable discussing their financial concerns. Such measures can help ensure that patients receive the necessary care without undue financial burden.^[44]^

## 8. Conclusion

This case underscores the significant challenges in diagnosing and managing disseminated Nocardia infections, particularly in patients with complex medical histories and immunocompromised states. The importance of early diagnosis, facilitated by advanced diagnostic tools such as next-generation sequencing (NGS), cannot be overstated. Tailored antimicrobial therapy, based on accurate pathogen identification, is crucial for effective treatment outcomes. Despite advancements in diagnostic modalities and therapeutic options, the mortality rates associated with Nocardia infections remain high, as highlighted by this case. This emphasizes the need for ongoing research to develop better management strategies and improve patient prognoses. Comprehensive care, including long-term follow-up, is essential to prevent recurrence and ensure optimal outcomes. The fatal outcome in this case also highlights the critical need for addressing financial barriers to healthcare access, which can significantly impact patient survival. Further studies are necessary to enhance our understanding and management of Nocardia infections, ultimately reducing their associated morbidity and mortality.

## Author contributions

**Conceptualization:** Yaolin Li.

**Data curation:** Weiguo Zhou.

**Methodology:** Xiayahu Li.

**Resources:** Kai Zhao.

**Writing – original draft:** Xiayahu Li.

**Writing – review & editing:** Yaolin Li.
